# RNA processing errors triggered by cadmium and integrator complex disruption are signals for environmental stress

**DOI:** 10.1186/s12915-019-0675-z

**Published:** 2019-07-16

**Authors:** Cheng-Wei Wu, Keon Wimberly, Adele Pietras, William Dodd, M. Blake Atlas, Keith P. Choe

**Affiliations:** 1 Department of Veterinary Biomedical Sciences, Western College of Veterinary Medicine, University of Saskatchewan, 52 Campus Drive, Saskatoon, SK S7N 5B4 Canada; 2 Department of Biology and Genetics Institute, University of Florida, Gainesville, FL 32611 USA; 3 Toxicology Centre, University of Saskatchewan, Saskatoon, SK S7N 5B3 Canada

**Keywords:** *Caenorhabditis elegans*, Cadmium, snRNA, RNA splicing, Stress response, Surveillance mechanism

## Abstract

**Background:**

Adaptive responses to stress are essential for cell and organismal survival. In metazoans, little is known about the impact of environmental stress on RNA homeostasis.

**Results:**

By studying the regulation of a cadmium-induced gene named *numr-1* in *Caenorhabditis elegans*, we discovered that disruption of RNA processing acts as a signal for environmental stress. We find that NUMR-1 contains motifs common to RNA splicing factors and influences RNA splicing in vivo. A genome-wide screen reveals that *numr-1* is strongly and specifically induced by silencing of genes that function in basal RNA metabolism including subunits of the metazoan integrator complex. Human integrator processes snRNAs for functioning with splicing factors, and we find that silencing of *C. elegans* integrator subunits disrupts snRNA processing, causes aberrant pre-mRNA splicing, and induces the heat shock response. Cadmium, which also strongly induces *numr-1*, has similar effects on RNA and the heat shock response. Lastly, we find that heat shock factor-1 is required for full *numr-1* induction by cadmium.

**Conclusion:**

Our results are consistent with a model in which disruption of integrator processing of RNA acts as a molecular damage signal initiating an adaptive stress response mediated by heat shock factor-1. When *numr-1* is induced via this pathway in *C. elegans*, its function in RNA metabolism may allow it to mitigate further damage and thereby promote tolerance to cadmium.

**Electronic supplementary material:**

The online version of this article (10.1186/s12915-019-0675-z) contains supplementary material, which is available to authorized users.

## Introduction

For most eukaryotic genes, introns must be spliced out of precursor mRNA (pre-mRNA) to produce mature mRNA transcripts ready for translation [1], a process that is essential to organismal development and homeostasis [2, 3]. Splicing is predominantly conducted in the nucleus by the spliceosome and associated RNA-binding proteins that form a dynamic, extremely complicated, and poorly understood macromolecular complex of small nuclear RNAs (snRNAs) and up to 300 distinct proteins [4]. In the last decade, an additional metazoan-specific complex of at least 14 proteins, named integrator, was discovered that indirectly promotes splicing by processing snRNAs into their functional forms [5–7].

Although mechanisms for regulating protein homeostasis during environmental stress have been studied in great detail [8], little is known about the regulation of RNA homeostasis during stress. Disruption of pre-mRNA processing has been observed during environmental stress [9, 10], and recent studies have revealed associations and causative relationships between splicing fidelity and aging, cancer, and myotonic dystrophy [11–13]. Splicing is also being explored as a therapeutic target [13]. A molecular understanding of how environmental stress affects RNA processing and adaptive cellular responses would provide novel insights into this core eukaryotic cell process with broad relevance to cell homeostasis and disease.

Responses to environmental stress almost always include transcriptional activation of genes encoding proteins that mitigate damage. However, translation of these stress response genes can be delayed if they contain introns and splicing is disrupted. In eukaryotes of all kingdoms, genes that are rapidly induced during stress contain few introns as a strategy to bypass splicing [14]. Genes encoding canonical heat shock protein (HSP) chaperone genes exemplify this strategy [15]. Coincidentally, HSPs have been shown to help restore RNA splicing during stress, presumably by promoting proper folding of splicing factor proteins [10, 15–17]. Other stress-responsive and intronless genes are poorly studied.

Using the genetic model nematode *Caenorhabditis elegans*, a previous study reported that a small intronless gene named *nuclear localized metal responsive-1* (*numr-1*) is highly induced by cadmium, a heavy metal and environmental contaminant [18–20]. Biochemical functions of NUMR-1 are unknown, but the protein was shown to localize to the nucleus and to promote *C. elegans* longevity and adult survival in cadmium [18]. NUMR-1 protein sequence has no resemblance to canonical metal-responsive metallothioneins that function as metal ion chelators [21].

We find that NUMR-1 contains motifs common in RNA-binding proteins and influences RNA splicing. We conducted a genome-wide RNA interference (RNAi) screen for regulators of *numr-1* and find that silencing of core splicing factors or integrator complex subunits strongly induces *numr-1* in the absence of cadmium. Cadmium or silencing of integrator complex subunits disrupts RNA splicing and processing of snRNAs suggesting a common molecular damage surveillance mechanism. Lastly, heat shock factor-1 (HSF-1), the guardian of the proteome, is activated by cadmium or integrator complex silencing and is required for full *numr-1* induction. Our results are consistent with a model where cadmium disrupts the processing of snRNAs by the integrator complex leading to HSF-1 activation of *numr-1* and HSPs as mechanisms to restore homeostasis.

## Results

### *numr-1* encodes an SR-like protein

**Fig. 1:**
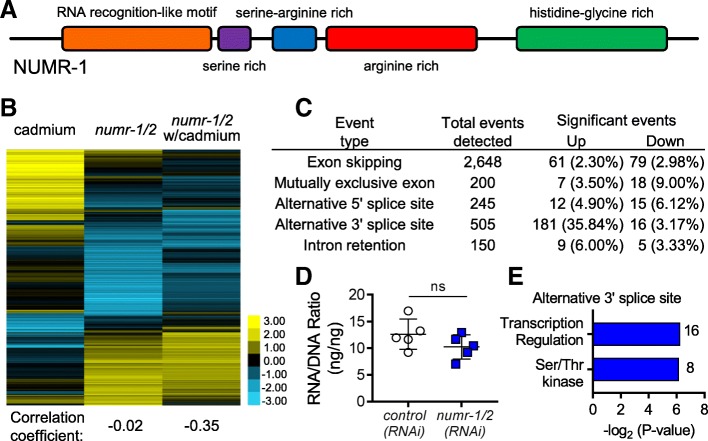
The *numr-1* gene encodes an SR-like protein that influences RNA splicing*.* **a** NUMR-1 resembles an SR-like protein with an N-terminal RNA recognition-like motif, serine- and arginine-rich regions, and a C-terminal histidine-glycine-rich region [22–24]. **b** Clustered heat map of log_2_ gene expression changes caused by 300 μM cadmium relative to no cadmium, *numr-1/2(RNAi)* relative to *control(RNAi)* on agar without cadmium, or *numr-1/2(RNAi)* relative to *control(RNAi)* on agar with 300 μM cadmium*.* Correlation coefficients relative to the first column of data are shown below. A list by gene is provided in Additional file 2: Table S1. **c** Summary of alternative splicing events caused by *numr-1/2(RNAi)* relative to *control(RNAi).* A list by gene is provided in Additional file 4: Table S3. Percentage of events that were significantly changed are provided in parentheses. **d** RNA to DNA ratios of worms fed with *control(RNAi)* or *numr-1/2(RNAi).* **e** Enriched categories within genes alternatively spliced by *numr-1/2(RNAi)*

Protein BLAST searches fail to identify NUMR-1 homologs outside of nematodes, which may be caused by high sequence divergence and stretches of low complexity. However, while inspecting primary sequence and secondary structure predictions, we identified motifs in NUMR-1 (Fig. 1a, Additional file 1: Figure S1) that resemble those found in serine-/arginine-rich (SR) proteins, a family of eukaryotic proteins with well-established roles in multiple steps of RNA metabolism [25]. Jpred4 secondary structure modeling [22] predicts a sequence of beta sheets and alpha helices at the N-terminus of NUMR-1 the same as RNA-recognition motifs (RRM) found in canonical SR proteins (Fig. 1a, Additional file 1: Figure S1). NUMR-1 also has serine- and arginine-rich regions that are common in SR proteins, and a C-terminus tail rich in histidine and glycine (Fig. 1a, Additional file 1: Figure S1). These features suggest that NUMR-1 might function in RNA metabolism.

### NUMR-1 influences RNA splicing

NUMR-1 was previously shown to reside in nuclei and promote longevity and survival of cadmium in adult worms, but nothing is known about its biochemical function [18]. The *numr-1* gene is immediately adjacent to a duplicate named *numr-2* that is controlled by the same promoter [18]. A deletion of both copies does not exist, but RNAi will target both because they are 100% identical at the nucleotide level. To explore the function of *numr-1/2* in RNA metabolism, we performed whole-genome RNA sequencing in worms fed *numr-1/2* dsRNA and compared it with worms fed control dsRNA with and without 12-h exposure to 300 μM cadmium.

A heat map of genes differentially expressed by cadmium or *numr-1/2(RNAi)* is shown in Fig. 1b with correlation coefficients relative to cadmium listed at the bottom. Cadmium changed the expression of 999 genes (771 up and 228 down) (Fig. 1b; Additional file 2: Table S1). As expected, genes at least fourfold upregulated by cadmium are enriched for functions in stress response, including drug metabolism, heat shock proteins, and c-type lectins [19] (Additional file 3: Table S2). RNAi of *numr-1/2(RNAi)* changed the expression of 1387 genes on control agar (518 up and 869 down) and 980 on agar with cadmium (538 up and 442 down) (Fig. 1b; Additional file 2: Table S1). Genes upregulated at least fourfold by *numr-1/2(RNAi)* with and without cadmium are only enriched for cuticle collagen, and there is no enrichment for genes downregulated by *numr-1/2(RNAi)* (Additional file 3: Table S2). Expression changes caused by *numr-1/2(RNAi)* were not correlated with expression changes caused by cadmium and only weakly negatively correlated on agar with cadmium (Fig. 1b). These results suggest that NUMR-1 does not function to regulate the expression of any specific type of genes associated with environmental stress responses.

More interestingly, *numr-1/2(RNAi)* led to 404 statistically significant changes to alternative splicing events (Fig. 1c, Additional file 4: Table S3). We did not detect a statistically significant decrease in total RNA to DNA ratios (Fig. 1d); with our sample size and standard deviations, we had a power of 0.8 to detect a 36% change. Of the five different types of alternate splicing events, significant changes in 3′ splice sites were the most numerous at 197 total and were the most enriched when normalized by the total number of events detected at 39%. Using DAVID analysis, we found that genes with significant changes in 3′ splice sites caused by *numr-1/2(RNAi)* were enriched for functions in transcription regulation and serine/threonine kinase (Fig. 1e). Examples of read maps for alternate 3′ splice site events are shown in Additional file 5: Figure S2.

### NUMR-1 promotes larval development in cadmium

Alternative mRNA splicing is essential for cell differentiation and tissue development [3]. To investigate the role of *numr-1* in development, we tested whether it influences larval growth of *C. elegans* in cadmium. Cadmium arrests larval development and decreases body size, and these effects were exacerbated by *numr-1/2* RNAi (Additional file 6: Figure S3). RNAi of *numr-1/2* did not affect body size under basal conditions indicating that the effects were specific to cadmium (Additional file 6: Figure S3). Therefore, NUMR-1 promotes larval development and growth in cadmium in addition to its previously reported role in promoting adult survival of cadmium and longevity [18].

### Silencing of spliceosome and integrator genes induces *numr-1*

**Fig. 2:**
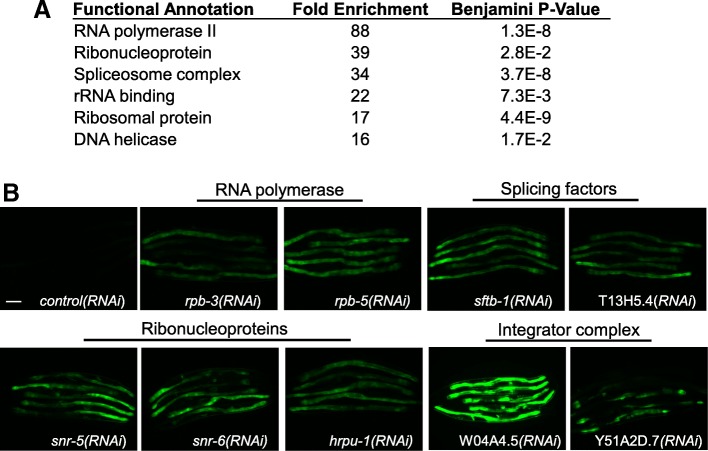
Silencing of genes functioning in RNA metabolism activates *numr-1.* **a** DAVID enrichment analysis of dsRNA clones that increased *numr-1p::GFP* fluorescence consistently. Complete list of RNAi hits shown in Additional file 8: Table S4. **b** Representative fluorescence micrographs of worms fed dsRNA targeting RNA metabolism genes that strongly activated the *numr-1p::GFP*; five worms are shown in each image; scale bar is 100 μm

Figure 1, Additional file 1: Figure S1, Additional file 5: Figure S2, and Additional file 6: Figure S3 provide evidence that NUMR-1 functions to influence RNA splicing and promotes tolerance of cadmium. To our knowledge, a stress-responsive gene that functions in RNA metabolism and splicing has not been described in metazoans [26, 27]. To gain insights into regulation of *numr-1*, we generated an integrated GFP reporter driven by the *numr-1* promoter (*numr-1p::GFP*). Fluorescence of *numr-1p::GFP* is increased by cadmium similar to endogenous *numr-1* as measured by qPCR (Additional file 7: Figure S4). We performed a genome-wide RNAi screen to identify genes that are required to maintain low basal *numr-1* expression and identified 170 dsRNA clones that activated *numr-1p::GFP* reproducibly (Additional file 8: Table S4). Using DAVID functional annotation analysis, we found a striking enrichment for RNA metabolic processes (Fig. 2a) [28]. Examples include RNA polymerase subunits, ribonucleoproteins, splicing factors, and subunits of the integrator complex (Fig. 2b).

**Fig. 3:**
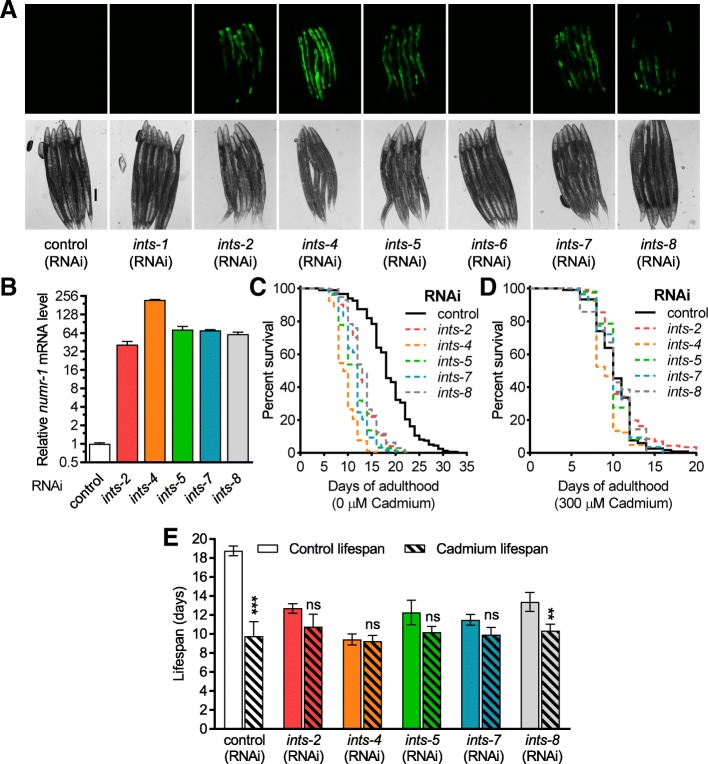
RNAi of integrator complex subunits induces *numr-1* and reduces survival of cadmium and longevity. **a** Representative fluorescence and DIC micrographs of worms fed dsRNA for *C. elegans* integrator complex subunits. Six worms are shown in each image, scale bar is 100 μm. N2 worms fed with control, *ints-2*, *ints-4*, *ints-5*, *ints-7*, or *ints-8* dsRNA were assessed for **b** fold changes in *numr-1* mRNA levels assessed by qPCR, **c** lifespan, and **d** survival of 300 μM cadmium from day 1 of adulthood. *N* = 4 replicates of 200–300 worms in **b** and two to three independent trials for (**c**, **d**). Statistics for individual trials in **c** and **d** are listed in Additional file 10: Table S6. In **b**, all integrator RNAi significantly increased *numr-1* mRNA with *P* < 0.001 compared to control dsRNA as determined by one-way ANOVA with Tukey post hoc tests. In **c**, all integrator RNAi significantly decreased the longevity of worms compared to control dsRNA as determined by the Log-rank test. **e** Median lifespans of individual trials from data in (**c**, **d**) and Additional file 10: Table S6. **P < 0.01 and ***P < 0.001 compared to corresponding control (0 μM cadmium) lifespan as determined by two-way ANOVA with Bonferroni post hoc tests

W04A4.5 is the dsRNA clone that caused the greatest increase in *numr-1p::GFP* fluorescence (Fig. 2b), and this gene encodes a homolog of human integrator complex subunit-4 (*ints-4*). Integrator mediates 3′ end processing of snRNA molecules that function in the spliceosome. The snRNA molecules are transcribed by RNA polymerase II but are not polyadenylated after transcription; instead, snRNA molecules are post-transcriptionally processed and cleaved at the 3′ end by the integrator [5, 29, 30]. Using BLAST, we found *C. elegans* homologs for 10/14 human integrator subunits (Additional file 9: Table S5), which match those recently reported by others [7]. *C. elegans ints* genes have been named based on their human homologs (Additional file 9: Table S5). RNAi of five out of seven of these integrator homologs available in our library increased *numr-1p::GFP* fluorescence (Fig. 3a), and these results were confirmed for endogenous *numr-1* with qPCR (Fig. 3b). Therefore, in the absence of exogenous stress, *numr-1* is induced by disruption of well-characterized RNA metabolism genes and the integrator complex.

### Disruption of integrator complex decreases lifespan

Loss of integrator homologs was previously reported to have detrimental effects on embryonic and larval development [31]. To further investigate the physiological functions of the integrator complex, we used RNAi to knockdown subunits identified from the RNAi screen to evaluate their effects on *C. elegans* lifespan and cadmium resistance. On control plates without cadmium, RNAi of each of the five integrator subunits identified in our screen reduced the lifespan of N2 worms by 5 to 8 days (Fig. 3c–e, Additional file 10: Table S6). Alternatively, RNAi of four out of five of these integrator subunits had no effect on survival in the presence of 300 μM cadmium (Fig. 3d, e, Additional file 10: Table S6). This lack of an additive effect raises the possibility that integrator complex RNAi and cadmium reduce lifespan by similar mechanisms.

### Cadmium alters 3′ processing of snRNAs

**Fig. 4:**
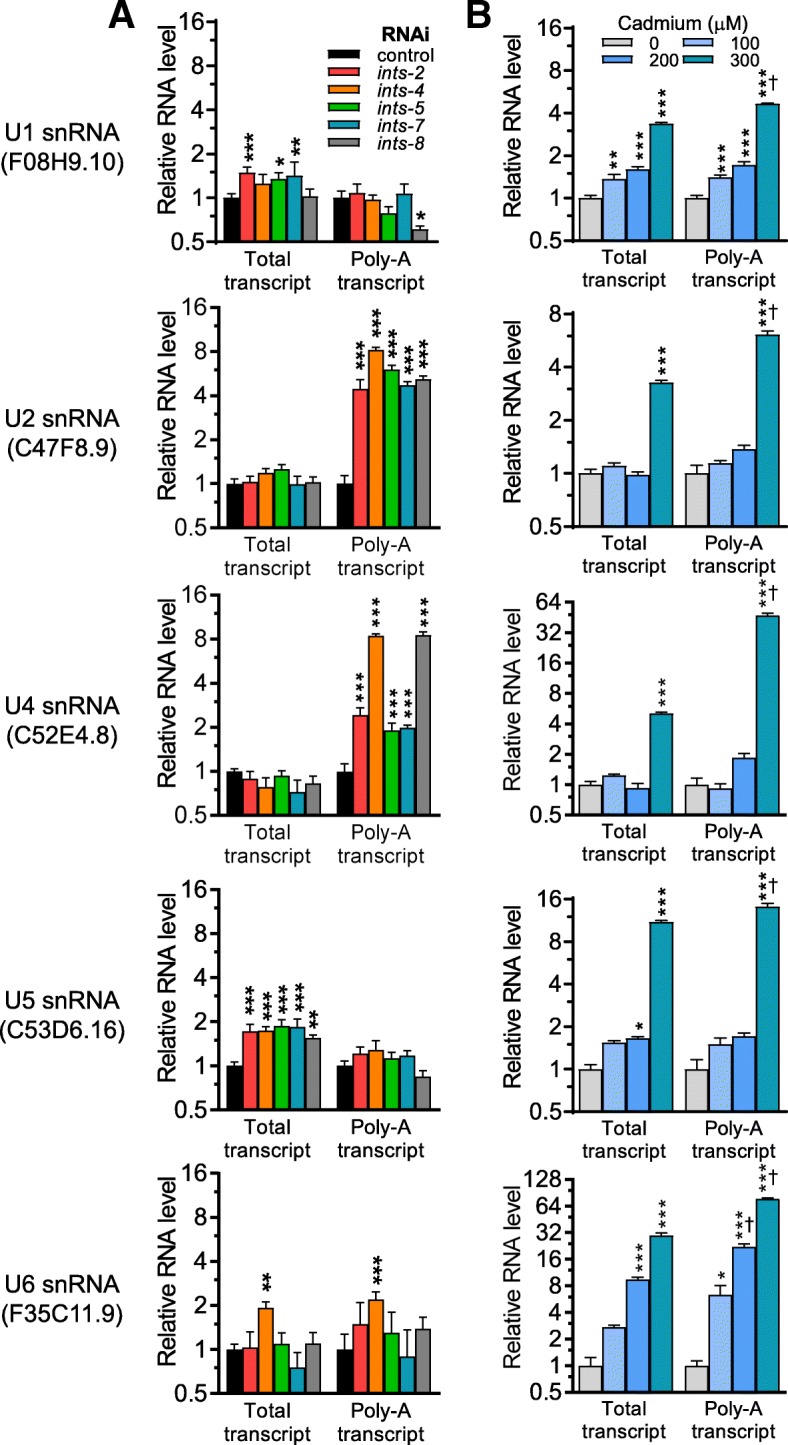
Cadmium disrupts snRNA 3′ processing. Fold change in total or polyadenylated (Poly-A) U1 snRNA (F08H9.10), U2 snRNA (C47F8.9), U4 snRNA (C52E4.8), U5 snRNA (C53D6.16), and U6 snRNA (F35C11.9) in N2 worms measured with qPCR. Worms were fed control, *ints-2*, *ints-4*, *ints-5*, *ints-7*, or *ints-8* dsRNA clones in **a** or exposed to 0, 100, 200, and 300 μM cadmium in **b***.* Random or oligo-dT primers were used to synthesize total or polyadenylated cDNA from each total RNA sample. *N* = 4 replicates of 200–300 worms. **a** *P < 0.05, **P < 0.01, or ***P < 0.001 compared to control dsRNA. **b** *P < 0.05, **P < 0.01, or ***P < 0.001 compared to 0 μM cadmium and ^†^*P* < 0.001 compared to the total transcript levels of the same cadmium concentration as determined by two-way ANOVA with Bonferroni post hoc tests

Data in Figs. 2 and 3 establish disruption of RNA metabolism and integrator as strong endogenous stimuli for *numr-1*. We next tested if disruption of RNA metabolism could be a signal for inducing *numr-1* when integrator subunits are silenced. Loss of integrator function in human cell lines, mice, and *Drosophila* causes the failure of transcription termination of snRNA and aberrant 3′ polyadenylation [29, 30, 32, 33]. A recent study also reported the same effect when *C. elegans* integrator homologs are disrupted [7]. To verify altered snRNA processing in *C. elegans* under our conditions, we silenced the five integrator complex genes that influence *numr-1* expression (Fig. 3) and measured snRNAs. To differentiate between total and polyadenylated (misprocessed) snRNAs, we synthesized two sets of cDNA from each RNA sample: one using random primers and one using oligo-dT primers [29]. Of the five *C. elegans* snRNA transcripts (U1, U2, U4, U5, and U6), polyadenylated U2 and U4 were increased the most by integrator homolog RNAi (Fig. 4a); there was little effect on the other snRNAs. These results confirm that 3′ processing of some snRNA by integrator is conserved in *C. elegans*.

We next tested if cadmium also disrupts snRNA processing. Exposure of worms to 300 μM cadmium for 24 h increased all total and polyadenylated snRNAs that we measured, but with a consistently larger effect on the latter (Fig. 4b). Importantly, this exposure to cadmium is not acutely lethal as worms survive for multiple days (Fig. 3d). These results indicate that cadmium and integrator RNAi both disrupt 3′ processing of snRNAs.

### Disruption of integrator complex and cadmium alter RNA splicing

**Fig. 5:**
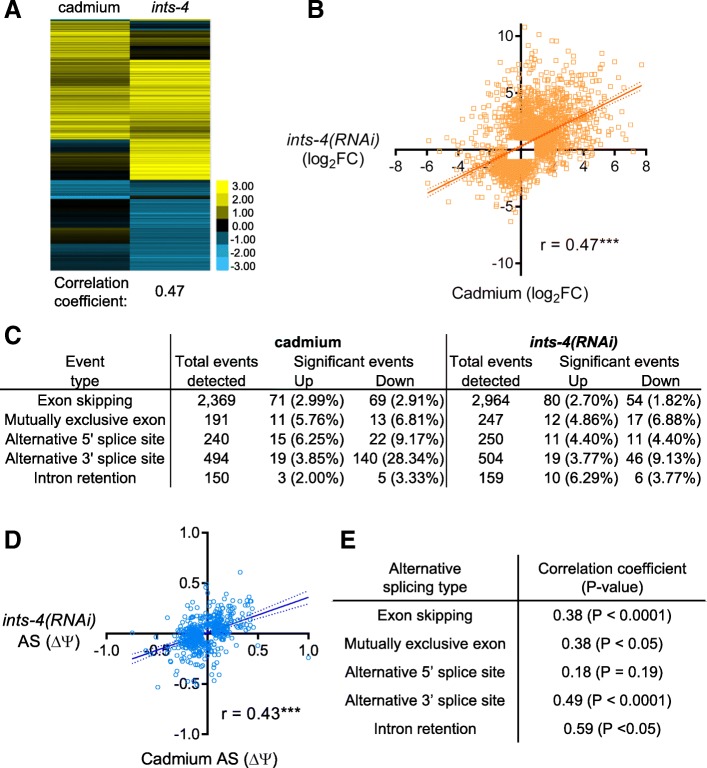
RNA splicing is disrupted by cadmium or RNAi of integrator complex. **a** Clustered heat map of log_2_ gene expression changes caused by 300 μM cadmium relative to no cadmium and *ints-4(RNAi)* relative to *control(RNAi)* on agar without cadmium*.* Correlation coefficient is shown below. A list by gene is provided in Additional file 2: Table S1. **b** Linear regression analysis of fold changes in panel **a**; ***P < 0.001 by linear regression *F* test. **c** Number of genes alternatively spliced within each category by cadmium or *ints-4(RNAi)*. Lists by gene are provided in Additional file 12: Table S7 and Additional file 13: Table S8. Percentage of events that were significantly changed are provided in parentheses. **d** Linear regression analysis of ΔΨ values for all significantly altered splicing events caused by *ints-4(RNAi)* or cadmium, ***P < 0.001 by linear regression *F* test. **e** Correlation coefficients and linear regression *P* values for each type of splicing event change caused by cadmium and *ints-4(RNAi)*

After snRNA transcripts are processed by the integrator complex, they interact with small nuclear ribonucleoproteins (snRNP) to form essential components of the spliceosome [1, 5]. Human cells with mutations in integrator complex subunits have genome-wide splicing defects consistent with malfunctioning snRNAs [34]. Given that cadmium and integrator disruption both alter 3′ processing of snRNAs (Fig. 4), we next tested for global changes in RNA processing using RNAseq. We used RNAi of *ints-4* because it caused the greatest induction of *numr-1/2* and misprocessing of *snRNAs* (Figs. 3 and 4); subunit-4 is also the most critical component of the *Drosophila* and human integrator complexes [35, 36]. RNAi of *ints-4* changed the expression of 1978 genes (1161 up and 817 down) (Additional file 2: Table S1). A clustered heat map of expression changes demonstrates striking similarity in the changes caused by 300 μM cadmium and *ints-4(RNAi)* (Fig. 5a; correlation coefficient is 0.47), particularly for genes upregulated. The 662 genes that were upregulated at least fourfold by *ints-4(RNAi)* are enriched for membrane proteins and heat shock proteins (Additional file 3: Table S2), the latter consistent with activation of protein homeostasis responses. There is no correlation between the gene expression changes caused by *ints-4(RNAi)* and *numr-1/2(RNAi)* (Additional file 11: Figure S5A-B).

Cadmium and *ints-4(RNAi)* caused 368 and 266 statistically significant alternative splicing events, respectively (Fig. 5c, Additional file 12: Table S7, Additional file 13: Table S8). To test if a correlation exists for the extent of alternative splicing, we performed linear regression analysis on relative percentage spliced in index scores (PSI, ΔΨ), which are a measure of the degree of alternative splicing*.* As shown in Fig. 5d, ΔΨ values for cadmium and *ints-4(RNAi)* alternate splicing events are highly correlated with a correlation coefficient of 0.43 (*P* < 0.001). We also analyzed the five different alternative splicing types separately and found significant ΔΨ correlation for four out of five splicing categories (Fig. 5e). In contrast, no correlation of ΔΨ values was found between spliced event changes caused by *numr-1/2(RNAi)* and *ints-4(RNAi)*, and ΔΨ values for *numr-1/2(RNAi)* and cadmium were only weakly negatively correlated (*r* = − 0.09; *P* = 0.02) (Additional file 11: Figure S5C-D). Taken together, results in Fig. 5 demonstrate that cadmium and integrator knockdown lead to overlapping changes in gene expression and mRNA splicing.

### HSF-1 regulates *numr-1*

**Fig. 6:**
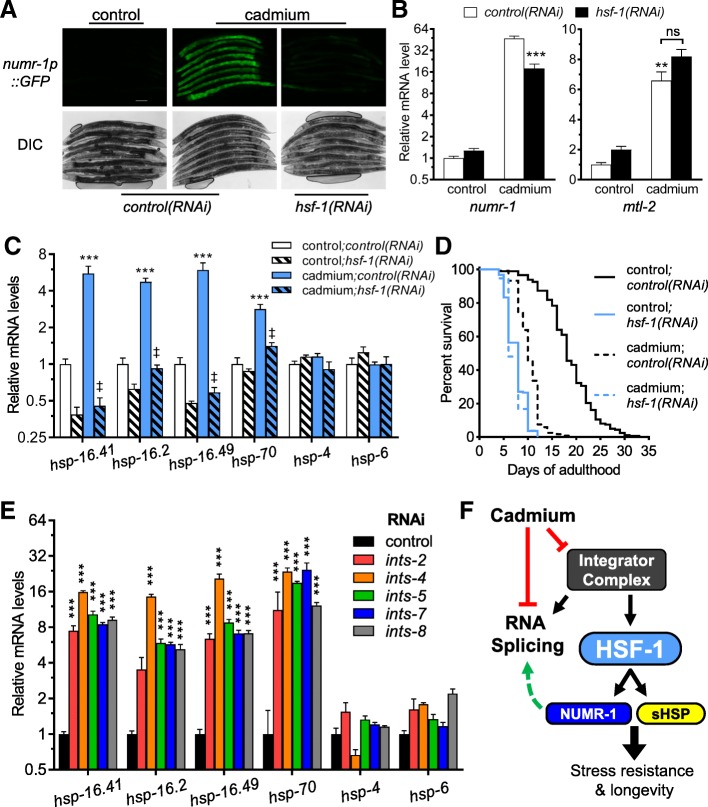
HSF-1 is required for full induction of *numr-1*. **a** Representative fluorescence and DIC micrographs of worms expressing *numr-1p::GFP* fed control or *hsf-1* dsRNA and treated with 0 (control) or 100 μM cadmium for 24 h. Eight worms are shown in each image; scale bar is 100 μm. Fold changes in *numr-1* (**b**), *mtl-2* (**b**), or heat shock protein gene (**c**) mRNA levels in N2 worms fed control or *hsf-1* dsRNA and treated with 0 (control) or 100 μM cadmium for 24 h as measured by qPCR. For **b** and **c**, *N* = 4 replicates of 200–300 worms. **P< 0.01 or ***P < 0.001 compared to control;*control(RNAi)* and ^‡^*P* < 0.001 compared to cadmium;*control(RNAi)* as determined by two-way ANOVA with Bonferroni post hoc tests. **d** Lifespan of N2 worms fed control or *hsf-1* dsRNA grown in the absence (control) or presence of 300 μM cadmium starting on day 1 of adulthood. Combined results are shown and statistics for individual trials are listed in Additional file 15: Table S9. The control data are the same as in Fig. 3c, d and listed in Additional file 4: Table S3. **e** Fold change in heat shock protein gene mRNAs in N2 worms fed with control, *ints-2*, *ints-4*, *ints-5*, *ints-7*, or *ints-8* dsRNA. ***P < 0.001 compared to control dsRNA as determined by two-way ANOVA with Bonferroni post hoc tests, *N* = 4 replicates with each replicate containing 200–300 worms. **f** Proposed model. Cadmium disrupts RNA splicing in part via RNA processing by the integrator complex as evident by a change in snRNA. Following integrator complex disruption, an *hsf-1*-dependent transcriptional response is activated to induce the expression of small heat shock protein genes and an RRM-like domain containing gene named *numr-1* that promotes RNA splicing, longevity, and stress resistance

To gain insights into the signaling mechanism that activates *numr-1*, we performed an RNAi screen to identify transcription factors required for *numr-1p::GFP* fluorescence. RNAi of three *C. elegans* transcription factors, *ceh-24* (*C. elegans* homeobox-24)*, fkh-6* (forkhead transcription factor family-6)*,* and *hsf-1* (heat shock factor-1), led to a significant reduction of *numr-1* mRNA (Additional file 14: Figure S6) with *hsf-1* the strongest and most consistent (Fig. 6a, b). Using qPCR, we next found that four cytosolic heat shock protein genes regulated by HSF-1 were induced by 100 μM cadmium indicating that the canonical heat shock response is activated in addition to *numr-1* (Fig. 6c); heat shock protein mRNA was also increased by 300 μM cadmium in RNAseq experiments (Additional file 2: Table S1, Additional file 3: Table S2). As others have reported, loss of *hsf-1* strongly decreases lifespan (Fig. 6d) [37]. Similar to *ints* subunit RNAi (Fig. 3), we found that 300 μM cadmium had no additive effect with *hsf-1(RNAi)* consistent with cadmium causing a type of cellular damage that overlaps with loss of HSF-1.

Heat shock protein genes of the endoplasmic reticulum (*hsp-4*) and mitochondrial (*hsp-6*) unfolded protein responses were not induced by 100 μM cadmium (Fig. 6c), suggesting specificity to the cytosolic response. Furthermore, RNAi of *hsf-1* did not reduce activation of the canonical heavy metal response gene *mtl-*2 (metallothionein-2) (Fig. 6b), which was previously shown to be regulated by transcription factor DAF-16/FOXO [38, 39]. Therefore, during cadmium exposure, HSF-1 is required for full activation of *numr-1* and the cytosolic heat shock response without affecting metal chelator or broader organelle-specific protein damage responses.

### Disruption of integrator complex induces the heat shock response

A previous study observed an increase in *hsp70p::GFP* fluorescence with RNAi of integrator complex subunit 4 (W04A4.5) [40] suggesting that, like cadmium, integrator disruption activates the heat shock response. As shown in Fig. 6e, RNAi of integrator subunits induces the same four cytosolic heat shock protein genes that were induced by cadmium (Fig. 6c). Taken together, these results are consistent with HSF-1 functioning to activate *numr-1* and cytosolic heat shock proteins when RNA metabolism is disrupted by cadmium or integrator silencing.

## Discussion

RNA metabolism is an extremely complex process with broad relevance to fundamental eukaryotic cell functions, pathogenesis, and aging. Relative to protein homeostasis, we know very little about how animal cells sense and respond to the disruption of RNA metabolism by environmental stress [10, 17, 26]. The intron-less gene *numr-1* was previously shown to encode a small nuclear protein that promotes longevity and is induced strongly by cadmium [18]. We find that NUMR-1 contains motifs similar to splicing factors, affects splicing *in vivo*, and promotes larval development in cadmium (Fig. 1, Additional file 1: Figure S1, Additional file 5: Figure S2, Additional file 6: Figure S3). Although stress-inducible splicing factors have not been previously identified in animals, a stress-inducible splicing factor named *SR34b* in the plant model *Arabidopsis* is also induced by cadmium, and *SR34b* mutation sensitizes plants to cadmium [41]. The presence of stress-inducible SR-like proteins in two different kingdoms is consistent with their fundamental importance to eukaryotic cell function during conditions that disrupt RNA metabolism.

Our genome-wide RNAi screen revealed that *numr-1* is strongly and specifically induced by disruption of endogenous RNA metabolic processes including the metazoan-specific integrator complex, which processes snRNA 3′ ends for functioning in the spliceosome [5, 6]. RNAi of integrator in *C. elegans* alters 3′ end processing of snRNA and RNA splicing in a manner similar to cadmium (Figs. 4 and 5). Because *numr-1* and the cytosolic heat shock response are adaptive responses to cadmium that are also activated by RNAi of integrator (Figs. 3 and 6), we propose that integrator processing of snRNAs may act as a surveillance mechanism for cadmium-induced molecular damage (Fig. 6f). Cadmium has broad effects in cells that are often attributed to oxidative stress [42]; direct effects on dsDNA structure have also been reported [43]. It remains to be determined how cadmium alters integrator processing of snRNAs, but it is conceivable that one or more of the protein-protein or protein-RNA interactions within integrator are altered directly by cadmium or redox changes, or indirectly by a signaling mechanism [6]. Interestingly, *numr-1* expression is far more sensitive to cadmium than other heavy metals suggesting specificity in the mechanism [18]. Given that integrator function is highly conserved [44], it could also be a potential target of cadmium in mammalian cells.

Other core cell processes are also used to detect stress. For example, disruption of protein translation is a signal for induction of osmotic response genes, protein miss-folding is a signal for the heat shock response, and loss of ribosome, mitochondria, and vesicle transport is a signal for detoxification responses [45–49]. This general strategy also extends to the tissue level as we recently identified a specific type of extracellular matrix disruption that induces three distinct stress responses in *C. elegans* [50].

Interestingly, RNAi of integrator subunit 4 had the largest effects on RNA splicing, *numr-1* expression, *hsp* expression, stress resistance, and longevity(Figs. 2, 3, and 6). Although some of these differences could be caused by variation in RNAi sensitivity, disruption of subunit 4 also has the largest effects on RNA processing and development in *Drosophila* and subunit 4 was recently shown to function as a scaffold for the catalytic core of human integrator complex [35, 36].

The full composition of integrator, interactions between subunits and accessory proteins, and regulation of integrator are active areas of research with relevance to transcription, cell cycle control, and a growing list of pathologies [6, 7]. Integrator mutation or silencing disrupts multiple steps of development and cell differentiation in *C. elegans*, *Drosophila*, zebrafish, and mouse cells [33, 51–54]. The human INTS6 (originally named deleted in cancer 1, DICE1) was first identified as a tumor suppressor in lung and squamous cell carcinomas and was recently shown to also act as a tumor suppressor in hepatocellular carcinomas [55–57]. INTS2 and INTS8 are also miss-expressed or mutated in many cancers [6]. Our study establishes cadmium regulation of *numr-1* in *C. elegans* as a paradigm for understanding interactions between integrator and the environment that is amenable to genetic analysis in a whole-animal context.

### HSF-1 and the cadmium stress response

Heat shock factors are highly conserved master regulators of heat shock protein chaperone genes under many conditions that perturb protein homeostasis. Cadmium was previously shown to activate heat shock factor in cultured mammalian cells [58–60]. As mentioned above, heat shock factors were also previously shown to respond to RNA splicing disruption by promoting expression of intronless *hsp* genes [15]. Our results demonstrate that the *C. elegans* cytosolic heat shock response is also activated by cadmium and is required for full induction of *numr-1* (Fig. 6 and Additional file 14: Figure S6). Unlike our results during exposure to cadmium (Fig. 6), *numr-1* is not induced by heat shock and is not dependent on HSF-1 under control or heat shock conditions in *C. elegans* [61]. The upstream regulatory region of *numr-1* does not contain a heat shock binding element [62]. Therefore, regulation of *numr-1* by HSF-1 is likely to be indirect and specific to conditions that target RNA metabolism.

HSF-1 has recently been shown to co-localize with pre-mRNA splicing factors in the nucleus of stressed cells within loci termed nuclear stress bodies (nSBs). These nSBs are thought to be sites of transcription and splicing of stress-induced transcripts [63, 64]. Interestingly, NUMR-1::GFP fusion proteins were previously shown to localize to nuclear loci with HSF-1::mCherry in *C. elegans* in response to cadmium [18]. It remains to be determined whether NUMR-1 functions to promote processing of transcripts at these loci.

## Materials and methods

### *C. elegans *strains

All *C. elegans* strains were cultured at 20 °C using standard methods [65]. The following strains were used in this study: N2 Bristol and QV151 *qvIs4[numr-1p::GFP]*.

### Analysis of NUMR-1 sequence

NUMR-1 secondary structure was predicted with JPred4 [22]. Integrator subunit BLAST searches were conducted within WormBase [31].

### RNAi experiments and genome-wide RNAi screen

A genome-wide RNAi screen was performed by feeding worms with *Escherichia coli* [HT115(DE3)] engineered to transcribe double-stranded RNA (dsRNA) homologous to *C. elegans* target genes using methods described previously [66, 67]. The complete ORFeome RNAi feeding library (Open Biosystems, Huntsville, AL) was screened in addition to clones supplemented from the original MRC genomic RNAi feeding library (Geneservice, Cambridge, UK). Briefly, synchronized L1 larvae of QV151 *C. elegans* were grown in liquid medium supplemented with dsRNA-producing bacteria for 3 days and screened manually for *numr-1p::GFP* fluorescence with a Zeiss Stemi SV12 microscope. Positive dsRNA clones were rescreened for three additional trials. dsRNA clones with positive results from all three trials are listed in Additional file 8: Table S4. To identify transcription factors that regulated *numr-1p::GFP*, *C. elegans* were grown in the same manner as described above using a transcription factor specific RNAi sub-library created from the ORFeome and MRC RNAi collections. After 3 days of dsRNA feeding, *C. elegans* were incubated with 100 μM of cadmium for 24 h to induce *numr-1p::GFP*, followed by manual fluorescent screening to identify transcription factor knockdowns that blocked cadmium-induced *numr-1p::GFP* activation. All subsequent RNAi experiments were performed with nematode growth medium (NGM) agar plates in the presence of 50 μg mL^−1^ carbenicillin and 0.2% β-lactose. RNAi feeding experiments on agar plates were all initiated at the synchronized L1 larval stage for 48–72 h before worms were used for subsequent experiments. The pPD129.36 (LH4440) clone was used as a control RNAi; it expresses a 202-bp dsRNA that is not homologous to any *C. elegans* genes.

### Fluorescent reporter analysis and PCR

To visualize the *C. elegans* fluorescent reporters under cadmium stress, worms were treated with either 100 μM of cadmium diluted in NGM buffer or NGM buffer alone as a control for 24 h at the L4/young adult (YA) stage. For RNAi experiments, worms were fed with appropriate dsRNA from L1 larvae to YA, or to the indicated worm age, before being imaged. Worms were mounted on 2% agarose pads with 5 mM levamisole and imaged using an Olympus BX60 microscope with a Zeiss AxioCam MRm camera fitted with either a GFP or a RFP filter (Center Valley, PA). To image whole worms that exceed the viewing plane under × 10 magnifications, multiple images were taken and merged into a single composite (Additional file 7: Figure S4).

Quantitative PCR (qPCR) assays were carried out as described previously using the delta-delta Ct method [68]. For all PCR assays, four replicate RNA samples were extracted per condition with each sample containing 100–300 synchronized worms. For samples from RNAi experiments, RNA was extracted from worms fed with the corresponding dsRNA from L1 larvae to young adult stage. For samples from cadmium exposure experiments, RNA was extracted from worms after exposure to cadmium for 24 h at the young adult stage. For analysis of snRNA transcripts, either random primers or oligo-dT primers were used to synthesize cDNA. All qPCR reactions were performed in 10 μL reaction volumes with a realplex^2^ (Eppendorf AG, Hamburg, Germany) with all data analyzed by normalization to the *rpl-2* gene as an internal reference. Primers used for qPCR are available on request.

### RNA/DNA assay

Worms were grown and treated the same as for whole-transcriptome RNA sequencing. For each biological replicate, five worms were picked to 10 μl of lysis buffer (5 mM Tris pH 8.0, 0.5% Triton X-100, 0.5% Tween 20, 0.25 mM EDTA, and 1 mg/ml proteinase K) and frozen at − 80 °C. Tubes containing frozen worms were placed immediately into a thermocycler and incubated 10 min at 65 °C and 2 min at 85 °C. Concentrations of RNA and DNA were determined with Promega Quantifluor dsDNA System (E2670) and Quantifluor RNA System (E3310). *N* = 5 biological replicates were used.

### Whole-transcriptome RNA sequencing

N2 worms were synchronized at the L1 larval stage and grown on RNAi agar plates seeded with either control, *numr-1/2*, or *ints-4* dsRNA-expressing bacteria until the late L3 stage. Worms were then collected in liquid NGM buffer and transferred to new RNAi agar plates containing either 0 or 300 μM cadmium for 12 h. For each of the six treatment groups, total RNA was extracted from three biological replicates of late L4 to young adult worms using an RNAqueous-Micro Total RNA isolation kit (ThermoFisher Scientific, AM1931) and sent to Novogene (Sacramento, CA) for oligo(dT) library construction, sequencing, mapping, and statistical analysis. HISAT2 was used to map sequences to the *C. elegans* reference genome [69]. Between 75 and 87 million clean reads and 11.4 and 13.1 Gbp were obtained from each sample; raw sequence data is available at Gene Expression Omnibus (GSE129970). Alternative splicing analysis was performed with rMATS using the total number of junction counts and reads on targets to quantify alternative splicing events [70, 71]. Gene Ontology enrichment was identified with DAVID functional analysis using high stringency, and we report Benjamini adjusted *P* values [28].

Differential expression analysis was carried out using DEGSeq, and we only considered transcripts detected in all treatment groups with changes of at least twofold. Because we are focused on environmental response and wanted to avoid differential gene expression changes caused indirectly by development, we also filtered out genes previously identified from ModEncode data to be enriched in one of the developmental stages potentially present in our samples (L4 larval, young adult, early embryo stages) [72]. To generate heat maps, differentially expressed genes were clustered with Gene Cluster 3.0 using correlation (uncentered) average linkage and mapped with Java Treeview 1.1.6r4.

### Lifespan, stress resistance, and developmental assays

For lifespan assays, synchronized L1 larvae were grown on NGM agar plates seeded dsRNA and scored for survival every 1 to 2 days starting at the third day of adulthood. For cadmium resistance assays, synchronized worms were grown under standard conditions from L1 to YA and then transferred onto appropriately seeded dsRNA NGM agar plates containing 300 μM cadmium. For both lifespan and stress resistance assays, adult worms were picked manually while gravid to avoid offspring. Worms with protruding gonad or intestine were censored, and worms were counted dead if they did not respond to gentle prodding with pick.

For developmental assays, synchronized L1 worms were fed dsRNA on NGM agar plates containing 0 or 250 μM of cadmium for 70 h before being imaged and quantified for body length using ImageJ. F_1_ offspring obtained from P_0_ worms fed with *numr-1/2* dsRNA were used for this assay to ensure silencing in early larvae. At least three independent trials were performed for all assays (with the exception of *ints-5* cadmium survival where only two trials were recorded), with the number of animals used in each experiment described in the corresponding supplementary table.

### Statistical analyses

With the exception of expression heat maps, data were analyzed and graphed using the Prism software 5.04 (La Jolla, CA) with Student’s *t* test performed when two means were compared, one-way ANOVA with Tukey’s post hoc test when multiple comparisons were performed with one factor, and two-way ANOVA with Bonferroni post hoc test in comparison over two factors. Longevity and survival assays were analyzed by the Log-rank test using the OASIS online statistic tool [73]. Linear regressions were analyzed by the *F* test. Statistical significance is indicated in each figure legend with *P < 0.05, **P < 0.01, and ^‡^/***P < 0.001. All *P* values for RNAseq analyses were corrected for FDR.

## Additional files

Additional file 1: **Figure S1.** NUMR-1 secondary protein structure prediction. (A) Jpred 4 analysis of NUMR-1 protein sequence reveals an N-terminal RRM-like domain from amino acids 23-87 defined by four beta strands (green) and two alpha helices (red) arranged in the order of β-α-β-β-α-β [22]. Similar structural arrangement of the RRM motif is found in a canonical SR protein and splicing factor, human serine- and arginine-rich splicing factor 2 (SRSF2) protein. (B) Complete amino acid sequence of the NUMR-1 protein highlighted with motifs identified in Fig. 1. (PDF 72 kb)

Additional file 2: **Table S1.** Differential gene expression. (XLSX 3482 kb)

Additional file 3: **Table S2.** DAVID gene enrichments. (XLSX 10 kb)

Additional file 4: **Table S3.** *numr-1* RNAi alternative splicing. (XLSX 796 kb)

Additional file 5: **Figure S2.** RNAi of *numr-1*/2 affects alternative 3′ splice site selection. Representative genome coverage tracks for alternative 3′ splice acceptor sites in *cdka-1,* M4.1, and R02F2.1 for control and *numr-1/2(RNAi)* samples. (PDF 186 kb)

Additional file 6: **Figure S3.** *numr-1* influences larval growth in cadmium. Effects of *numr-1/2(RNAi)* on body length of *C. elegans* grown with 0 or 250 μM cadmium assessed 70 h after hatching from L1 larvae. L1 *numr-1/2(RNAi)* worms were the F_1_ population obtained from P_0_ that were fed with *numr-1/2* RNAi. ***P < 0.001 compared to *control(RNAi)* as determined by Student’s *t* test, with 72–75 animals measured per condition from 3 trials. Scale bar is 100 μm. (PDF 79 kb)

Additional file 7: **Figure S4.** The *numr-1* GFP reporter and endogenous mRNA are activated by cadmium. (A) *numr-1p::GFP* representative fluorescence and DIC micrographs of worms exposure to control NGM buffer or NGM buffer with 100 μM of cadmium for 24 h at the L4/YA stage. Six worms are shown in each image, scale bar is 100 μm. (B) Relative mRNA levels of the *numr-1* gene in control and 100-μM-cadmium-treated N2 worms for 24 h as assessed by qPCR. This data is replotted from Fig. 6b. *N* = 4 replicates of 200–300 worms. ***P < 0.001 compared to control as determined by Student’s *t* test. (PDF 101 kb)

Additional file 8: **Table S4.** Gene dsRNAs that induce *numr-1p*::GFP. (XLSX 20 kb)

Additional file 9: **Table S5.** Integrator complex subunit homology. (XLSX 9 kb)

Additional file 10: **Table S6.** Integrator longevity and cadmium survival. (XLSX 13 kb)

Additional file 11: **Figure S5.** *ints-4* and *numr-1* gene expression and alternative splicing effects are not correlated. (A) Clustered heat map of log_2_ gene expression changes caused by *numr-1/2(RNAi)* and *ints-4(RNAi)* relative to *control(RNAi)* without cadmium*.* Correlation coefficient is shown below. A list by gene is provided in Additional file 2: Table S1. (B) Linear regression analysis of fold changes shown in panel A*.* Linear regression analysis of ΔΨ values for all significantly altered splicing events caused by *numr-1/2 (RNAi)* or *ints-4(RNAi)* in (C) and by *numr-1/2(RNAi)* and cadmium in (D)*. (PDF 109 kb)*

Additional file 12: **Table S7.** Cadmium exposure alternative splicing. (XLSX 757 kb)

Additional file 13: **Table S8.** *ints-4* RNAi alternative splicing. (XLSX 893 kb)

Additional file 14: **Figure S6.** Transcription factors required for *numr-1* activation. (A) Workflow of the transcription factor RNAi screen. (B) Representative fluorescence micrographs of worms fed dsRNA that inhibited cadmium-induced *numr-1p::GFP* activation, 8 worms are shown in each image, and scale bar is 100 μm. Images shown for control and *hsf-1(RNAi)* are the same as Fig. 6a. (C) Gene names and descriptions of transcription factors required for full *numr-1* induction. (D) qPCR analysis of *numr-1* mRNA levels fed with control, *ceh-24*, *fkh-6*, and *hsf-1* dsRNA under control and cadmium exposed conditions. ***P < 0.001 as determined by two-way ANOVA with Bonferroni post hoc tests, *N* = 4 replicates with each replicate containing 200–300 worms. (PDF 423 kb)

Additional file 15: **Table S9.** *hsf-1* longevity and cadmium survival. (XLSX 8 kb)

## Data Availability

All datasets supporting the conclusions of the manuscript are included within the article and its additional files. Raw data used to generate individual graphs in this manuscript are available at https://figshare.com/s/9cae6f74962d85ceab6a [74]. The accession number for the RNA-sequencing data presented in this paper is GEO: GSE129970 https://www.ncbi.nlm.nih.gov/geo/query/acc.cgi?acc=GSE129970 [75].
