# Supplementary material for: RNA processing errors triggered by cadmium and integrator complex disruption are signals for environmental stress
Source: BMC Biol. 2019 Jul 16;17:56. doi: 10.1186/s12915-019-0675-z (PMC6631800; doi:10.1186/s12915-019-0675-z)

A

NUMR-1

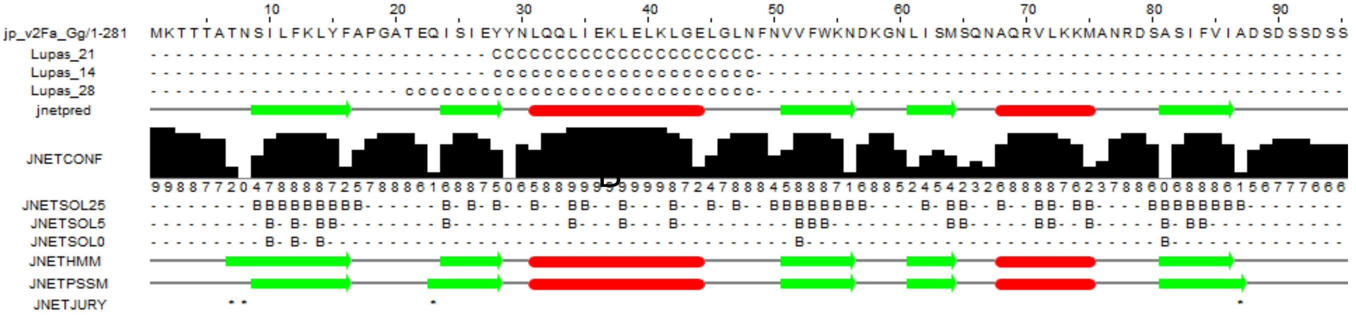

SRSF2 serine and arginine rich splicing factor 2

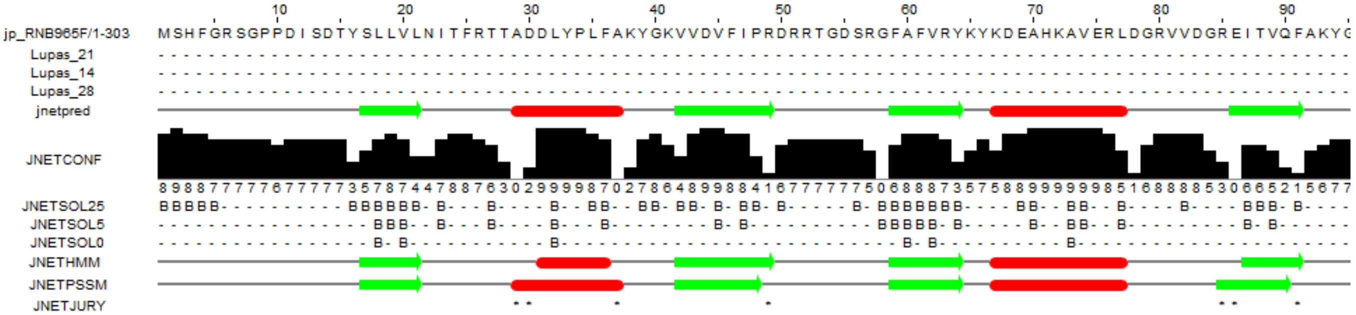

The annotation bars below the alignment are as follows:

•Lupas_21, Lupas_14, Lupas_28

Coiled-coil predictions for the sequence. These are binary predictions for each location.

•JNetPRED

The consensus prediction - helices are marked as red tubes, and sheets as dark green arrows.

•JNetCONF

The confidence estimate for the prediction. High values mean high confidence. prediction - helices are marked as red tubes, and sheets as dark green arrows.

•JNetHMM

HMM profile based prediction - helices are marked as red tubes, and sheets as dark green arrows.

•JNETPSSM

PSSM based prediction - helices are marked as red tubes, and sheets as dark green arrows.

•JNETJURY

A '' in this annotation indicates that the JNETJURY was invoked to rationalize significantly different primary predictions.*

B

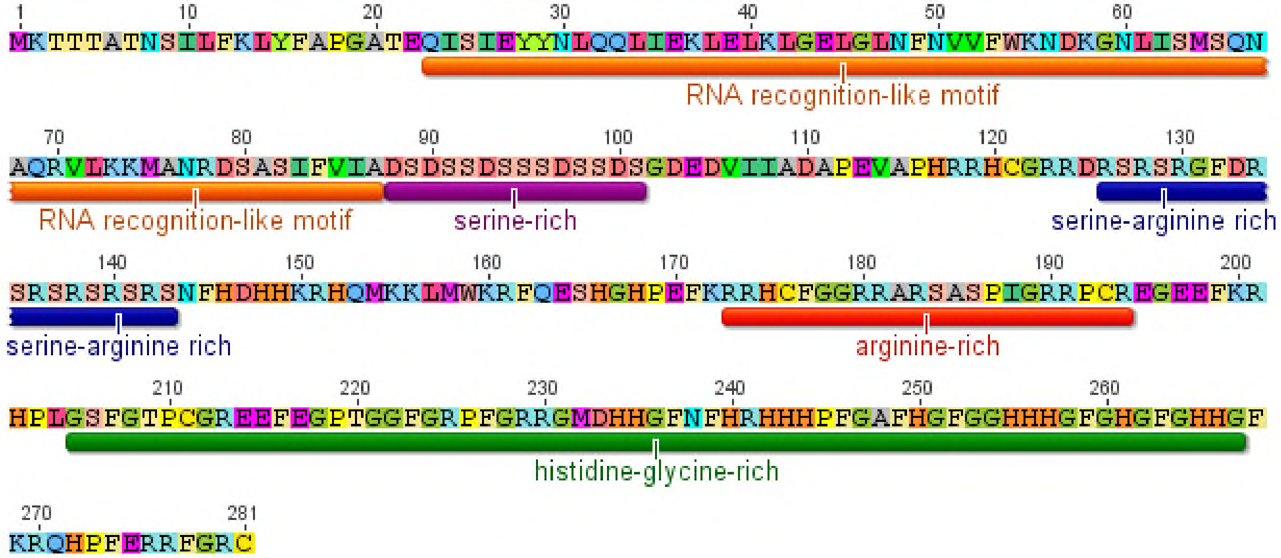

Supplement: Supplementary file 1 — Figure S1. NUMR-1 secondary protein structure prediction. (A) Jpred 4 analysis of NUMR-1 protein sequence reveals an N-terminal RRM-like domain from amino acids 23-87 defined by four beta strands (green) and two alpha helices (red) arranged in the order of β-α-β-β-α-β [22]. Similar structural arrangement of the RRM motif is found in a canonical SR protein and splicing factor, human serine- and arginine-rich splicing factor 2 (SRSF2) protein. (B) Complete amino acid sequence of the NUMR-1 protein highlighted with motifs identified in Fig. 1. (PDF 72 kb) [file 12915_2019_675_MOESM1_ESM.pdf]
