# Supplementary figures and images for: RNA processing errors triggered by cadmium and integrator complex disruption are signals for environmental stress
Source: BMC Biol. 2019 Jul 16;17:56. doi: 10.1186/s12915-019-0675-z (PMC6631800; doi:10.1186/s12915-019-0675-z)

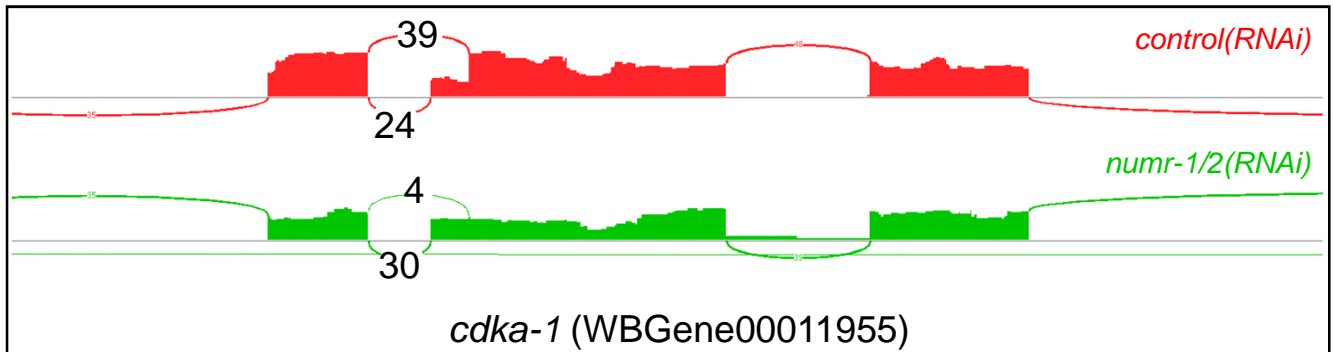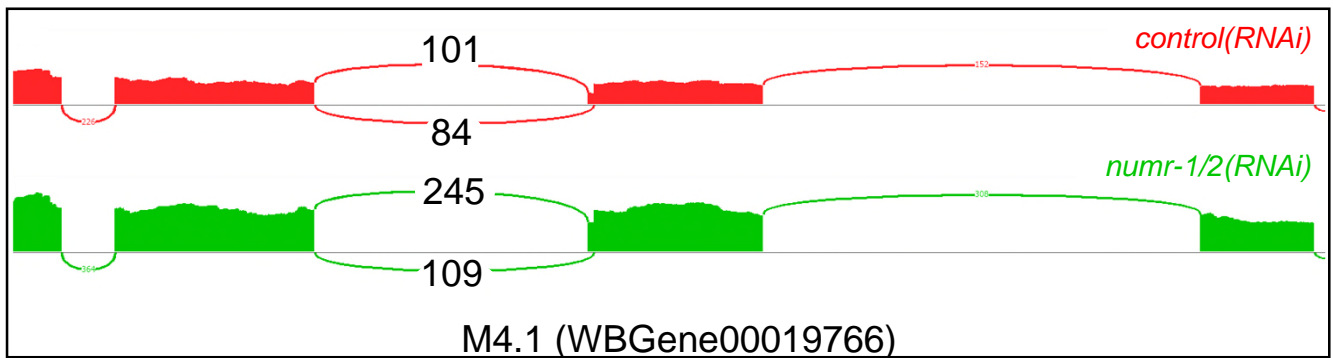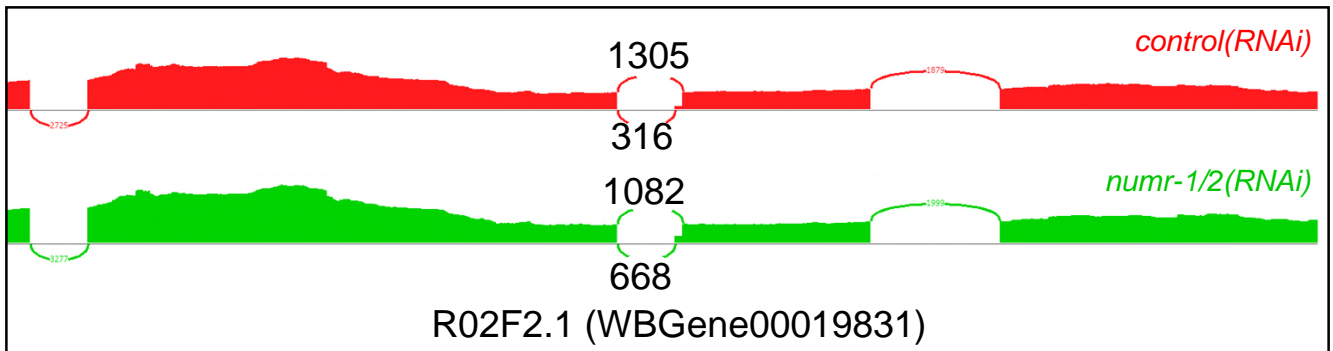

Supplement: Supplementary file 5 — Figure S2. RNAi of numr-1/2 affects alternative 3′ splice site selection. Representative genome coverage tracks for alternative 3′ splice acceptor sites in cdka-1, M4.1, and R02F2.1 for control and numr-1/2(RNAi) samples. (PDF 186 kb) [file 12915_2019_675_MOESM5_ESM.pdf]

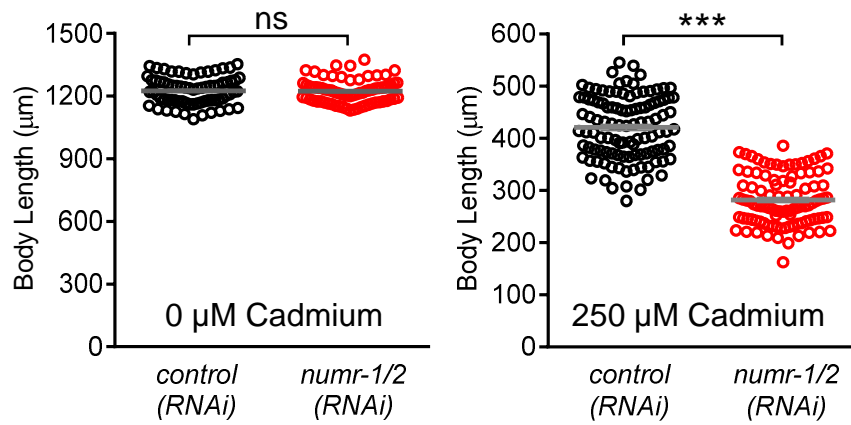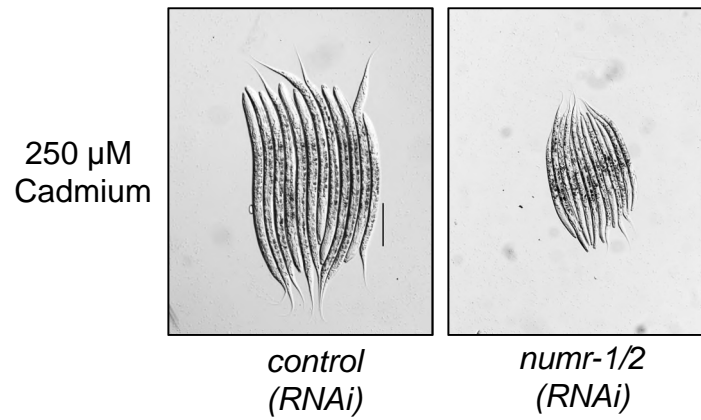

Supplement: Supplementary file 6 — Figure S3. numr-1 influences larval growth in cadmium. Effects of numr-1/2(RNAi) on body length of C. elegans grown with 0 or 250 μM cadmium assessed 70 h after hatching from L1 larvae. L1 numr-1/2(RNAi) worms were the F1 population obtained from P0 that were fed with numr-1/2 RNAi. ***P < 0.001 compared to control(RNAi) as determined by Student’s t test, with 72–75 animals measured per condition from 3 trials. Scale bar is 100 μm. (PDF 79 kb) [file 12915_2019_675_MOESM6_ESM.pdf]

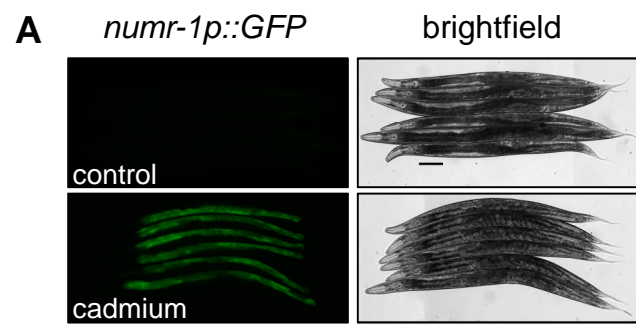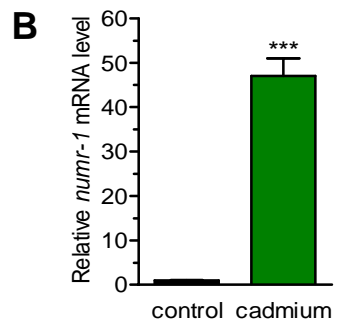

Supplement: Supplementary file 7 — Figure S4. The numr-1 GFP reporter and endogenous mRNA are activated by cadmium. (A) numr-1p::GFP representative fluorescence and DIC micrographs of worms exposure to control NGM buffer or NGM buffer with 100 μM of cadmium for 24 h at the L4/YA stage. Six worms are shown in each image, scale bar is 100 μm. (B) Relative mRNA levels of the numr-1 gene in control and 100-μM-cadmium-treated N2 worms for 24 h as assessed by qPCR. This data is replotted from Fig. 6b. N = 4 replicates of 200–300 worms. ***P < 0.001 compared to control as determined by Student’s t test. (PDF 101 kb) [file 12915_2019_675_MOESM7_ESM.pdf]

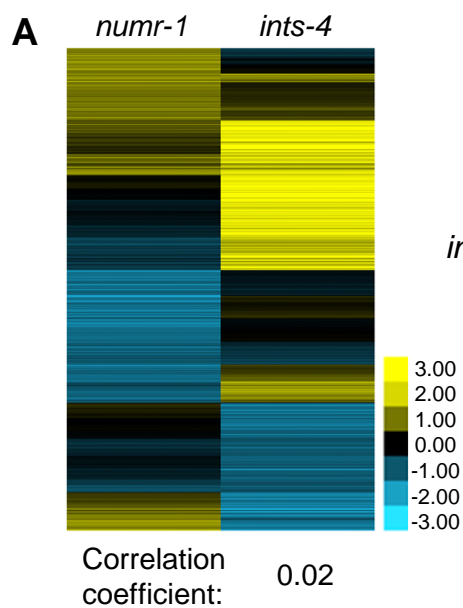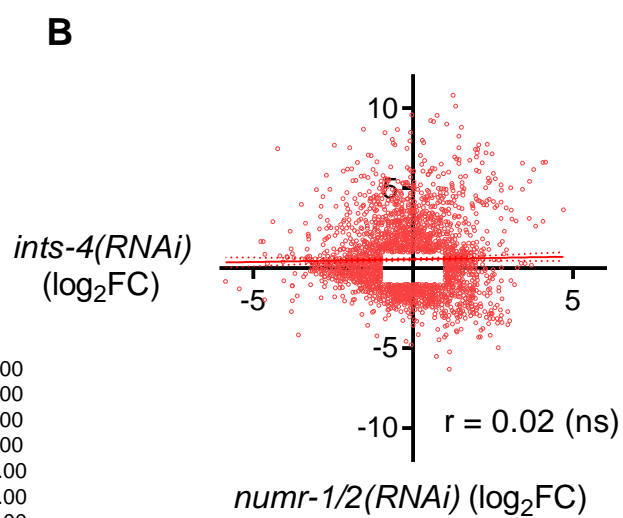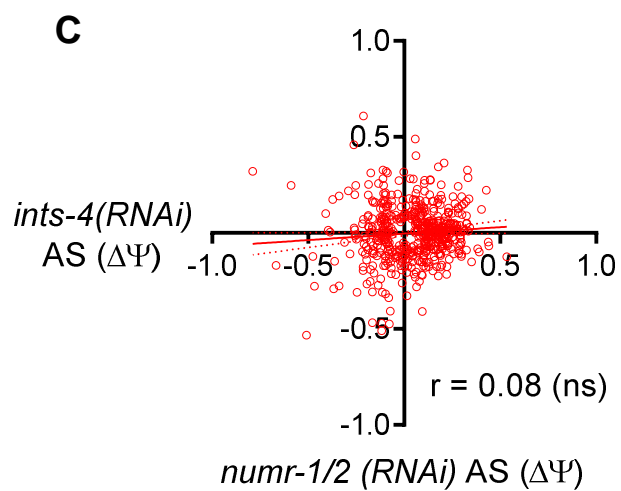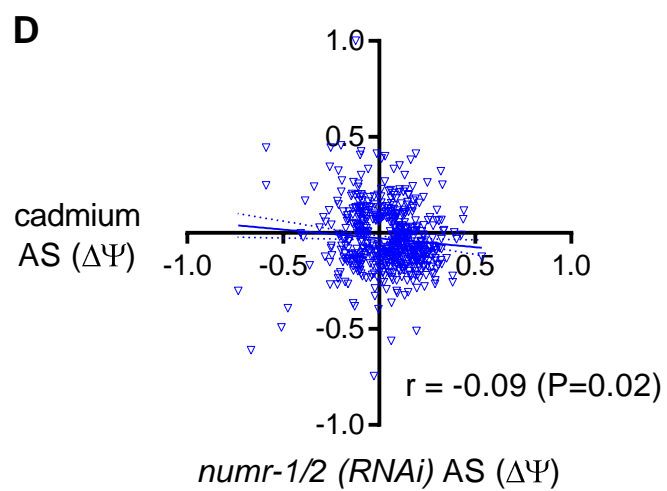

Supplement: Supplementary file 11 — Figure S5. ints-4 and numr-1 gene expression and alternative splicing effects are not correlated. (A) Clustered heat map of log2 gene expression changes caused by numr-1/2(RNAi) and ints-4(RNAi) relative to control(RNAi) without cadmium. Correlation coefficient is shown below. A list by gene is provided in Additional file 2: Table S1. (B) Linear regression analysis of fold changes shown in panel A. Linear regression analysis of ΔΨ values for all significantly altered splicing events caused by numr-1/2 (RNAi) or ints-4(RNAi) in (C) and by numr-1/2(RNAi) and cadmium in (D). (PDF 109 kb) [file 12915_2019_675_MOESM11_ESM.pdf]
