# Supplementary material for: RNA processing errors triggered by cadmium and integrator complex disruption are signals for environmental stress
Source: BMC Biol. 2019 Jul 16;17:56. doi: 10.1186/s12915-019-0675-z (PMC6631800; doi:10.1186/s12915-019-0675-z)

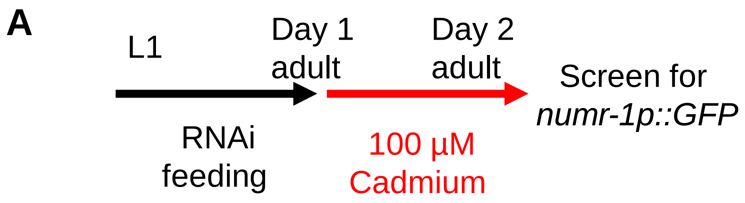

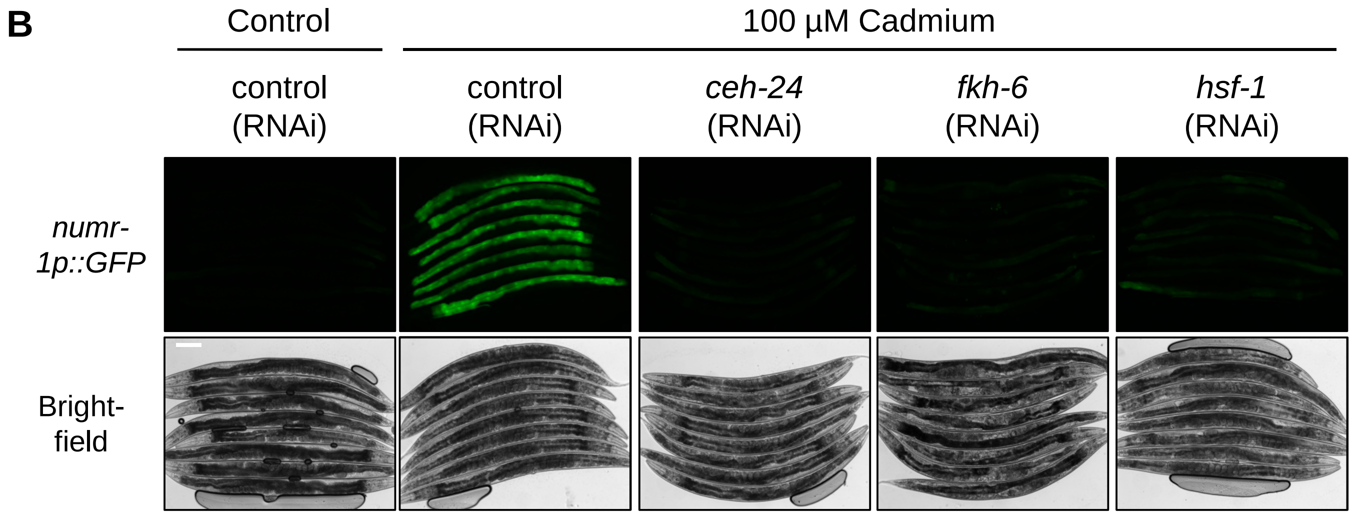

C

Gene	Description
ceh-24	Ortholog of human NK2-1 and 4 (NK2 homeobox 1 and 4)
fkh-6	Ortholog of human FOXE3 (forkhead box E3)
hsf-1	Ortholog of human HSF1 (heat shock factor 1)

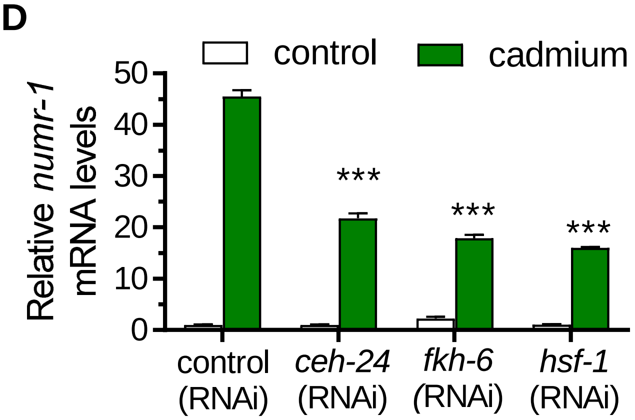

Supplement: Supplementary file 14 — Figure S6. Transcription factors required for numr-1 activation. (A) Workflow of the transcription factor RNAi screen. (B) Representative fluorescence micrographs of worms fed dsRNA that inhibited cadmium-induced numr-1p::GFP activation, 8 worms are shown in each image, and scale bar is 100 μm. Images shown for control and hsf-1(RNAi) are the same as Fig. 6a. (C) Gene names and descriptions of transcription factors required for full numr-1 induction. (D) qPCR analysis of numr-1 mRNA levels fed with control, ceh-24, fkh-6, and hsf-1 dsRNA under control and cadmium exposed conditions. ***P < 0.001 as determined by two-way ANOVA with Bonferroni post hoc tests, N = 4 replicates with each replicate containing 200–300 worms. (PDF 423 kb) [file 12915_2019_675_MOESM14_ESM.pdf]
